# Effects of alamandine on monocrotaline-induced pulmonary hypertension in rats

**DOI:** 10.22038/IJBMS.2023.74865.16254

**Published:** 2024

**Authors:** Ava Soltani Hekmat, Freshteh Amini, Kazem Javanmardi

**Affiliations:** 1 Department of Physiology, Fasa University of Medical Sciences, Fasa, Iran

**Keywords:** Alamandine, Hypertension, Monocrotaline, Oxidative stress, Pulmonary, Renin–angiotensin system

## Abstract

**Objective(s):**

Pulmonary arterial hypertension (PAH) is a severe and often fatal disease that is associated with oxidative stress and inflammation. Alamandine, a component of the renin-angiotensin system, known for its antioxidative, anti-inflammatory, and antifibrotic effects, has been investigated in this study to determine if it has protective effects against PAH induced by monocrotaline (MCT) and if these effects are associated with oxidative stress, inflammatory factors, and inducible nitric oxide synthase (iNOS).

**Materials and Methods:**

Rats were administered MCT (40 mg/kg) on day 0 and then received alamandine (50 mg/kg/day) via mini-osmotic pumps for 21 days starting one day later. Hemodynamic parameters, electrocardiograms, superoxide dismutase (SOD), catalase (CAT), malondialdehyde (MDA), inflammatory cytokines (TNF-α, IL-1β, and NF-κB), iNOS, and MrgD receptor expression in lung tissue were evaluated at the end of the 21-day period. The MrgD receptor was quantified through immunofluorescent staining, and the histopathology of lung tissues was evaluated using hematoxylin and eosin staining.

**Results:**

The results showed that alamandine treatment significantly improved hemodynamic parameters, oxidative stress markers, inflammatory factors, and electrocardiographic data. Furthermore, treatment with alamandine decreased the levels of iNOS. Additionally, alamandine treatment decreased the expression levels of MrgD receptors in the lung tissue of MCT-induced PAH.

**Conclusion:**

In summary, this study indicates that alamandine has protective effects against monocrotaline-induced PAH, and these effects may be attributed to the inhibition of oxidative stress, inflammatory parameters, and iNOS.

## Introduction

Pulmonary arterial hypertension (PAH) is characterized by elevated blood pressure in the pulmonary arteries, leading to increased mortality risks. The contemporary definition of PAH has been reshaped by recent outcome data, placing emphasis on early detection. According to the updated criteria, PAH is diagnosed in individuals with a mean pulmonary artery pressure exceeding 20 mmHg, as confirmed by right heart catheterization (1). The growing prevalence of PAH is influenced by an aging population, increased incidence of heart and lung diseases, and improved survival rates due to targeted therapies (2).

A hallmark of PAH is the proliferation of vascular wall cells and the remodeling of precapillary arteries, causing elevated pulmonary artery pressure and eventual right ventricular failure (3, 4). This condition is underlined by various degrees of pulmonary arterial vessel remodeling, resulting in increased mean pulmonary artery pressure, pulmonary vascular resistance, and subsequently, right ventricular hypertrophy (RVH) and failure (4, 5).

The pathology of PAH is multifaceted, rooted in endothelial dysfunction, which manifests as an imbalance between vasoconstrictive and vasodilatory factors such as endothelin and nitric oxide (NO). This imbalance instigates pulmonary vasoconstriction and vascular remodeling, leading to a persistent rise in pulmonary vascular resistance (6). Furthermore, cytokines play a pivotal role in PAH’s pathogenesis (7).

Inflammation stands out as a fundamental hallmark in the pathophysiology of PAH. Clinical and foundational research consistently underlines its significance in PAH development (8). One of the manifestations of this is seen in the fact that PAH is frequently a complication of autoimmune diseases, including systemic sclerosis, HIV infection, and schistosomiasis. Moreover, perivascular inflammation is commonly seen in various PAH types, as evidenced by cellular markets such as the infiltration of mast cells, T and B lymphocytes, and macrophages around the vessels (9). Another telltale sign is the elevated levels of cytokines like tumor necrosis factor (TNF), interleukin-1 (IL-1), and interleukin-6 (IL-6) in both lung tissue and blood (10). These cytokines are more than just markers; they potentially serve diagnostic and prognostic purposes in PAH. For instance, elevated serum IL-1β levels in PAH patients have been directly linked with unfavorable outcomes (8, 11). 

On a parallel front, oxidative stress has emerged as a pivotal factor in PAH’s development, with multiple studies supporting this connection (12). Central to this are the disturbances observed in the reactive oxygen species and NO signaling pathways. An upset in the oxidant/antioxidant equilibrium adversely affects vascular tone, precipitating the abnormal activation of antiapoptotic and mitogenic routes. This, in turn, drives unchecked cell growth and culminates in vasculature obliteration (13). In experimental setups, interventions using antioxidants have demonstrated potential protective effects against PAH (13). 

While the introduction of advanced pharmaceuticals like prostanoids, endothelin receptor antagonists, and phosphodiesterase-5 inhibitors has revolutionized PAH prognosis, the challenge remains. The persistently high morbidity and mortality rates underscore an unmet need (14). There is an imperative demand for innovative PAH therapeutic strategies that are intricately woven with its detailed pathophysiology.

One such potential avenue is the renin-angiotensin system (RAS). All major RAS components are expressed in lung tissues, making it pivotal for lung pathophysiology (15). Alamandine is a new member of the angiotensin family that has exhibited significant cardioprotective effects in rats treated with isoproterenol. This peptide is similar to Ang-(1–7) and can bind to the Mas-related G-coupled receptor known as member D (MrgD) (15, 16). Alamandine has both antifibrotic and anti-inflammatory effects (17, 18).It increases antioxidant expression in ventricles exposed to ischemia-reperfusion injury (19) and reduces pro-inflammatory factors induced by aortic constriction, such as TNF-α and IL-1α, in mice (20). In a study on an animal model of sepsis induction by polysaccharides in C57BL6/J mice, the plasma and tissue levels of IL-1B and TNFα increased. Administration of alamandine reduced the levels of inflammatory cytokines and apoptosis in cardiac tissues (21).

Based on the antioxidant and anti-inflammatory effects of alamandine, we hypothesize that the activation of the protective arm of the RAS by alamandine may attenuate various characteristics of PAH in a rat pulmonary hypertension model through biochemical, hemodynamic, and histopathological studies. 

## Materials and Methods

Male Sprague-Dawley rats, aged 6–8 weeks (weight = 200–220 g), were housed in groups of five in a temperature-controlled room maintained at 22±2 °C with a 12:12-hr light-dark cycle, lights on at 7:00 AM. The animals were provided free access to standard laboratory chow and water. All experimental protocols conformed to the National Institutes of Health Guide for the Care and Use of Laboratory Animals and were approved by Fasa University of Medical Sciences, Iran, with the ethical code (IR.FUMS.AEC.1401.010), obtained on September 7, 2022. A total of 28 rats were used in this study, divided into 4 groups of 7 rats each.

For biochemical and hemodynamic studies, rats were anesthetized via intraperitoneal ketamine (70 mg/kg) and xylazine (30 mg/kg). The anesthesia level was evaluated using the toe-pinch reflex.

***Experimental protocols***

In this study, we used monocrotaline (MCT) for induction of pulmonary hypertension. The advantages of the MCT model are its technical simplicity, reproducibility, and relatively low cost (5). Subjects were randomly allocated into various experimental and control groups. To ensure impartiality and accuracy in results, all stages of the study, including selection, treatment, and outcome assessment, were conducted in a blinded manner.‎ Rats were divided randomly into the following groups:

Control Group (Group I): In the control group, saline was administered via mini-osmotic pumps (model 2006; ALZET Osmotic Pumps, CA, USA) at an infusion rate of 0.15 μl/hr, mirroring the administration method employed for the experimental groups. Additionally, rats in this group received an intraperitoneal (IP) injection of saline to maintain consistency with the injection protocol used in the other groups.

MCT Group (Group II): Rats in this group received an IP injection of 40 mg/kg MCT (22) on day 0. Due to the restrictions imposed by the local animal ethics and welfare committee rules, experiments involving rats exposed to 40 mg/kg MCT were limited to 25 days. Therefore, right ventricular pressure and other parameters were measured on day 22 after MCT injection, as this was the final day of the experimental period to avoid the risk of end-stage heart failure and premature death observed in previous experiments (22).

Ala Group (Group III): Rats in this group were administered alamandine (Phoenix Pharmaceuticals Inc., CA, USA) for 21 days via mini-osmotic pumps with an infusion rate of 0.15 μl/hr (approximately 50 μg alamandine/kg/day) (23–26)starting from day 1.

MCT+Ala Group (Group IV): In this group, rats received alamandine via mini-osmotic pumps for 21 days (approximately 50 μg alamandine/kg/day), starting from day 1, and an IP injection of MCT in normal saline (40 mg/kg) on day 0.

***Measurements of right ventricular systolic pressure and weight***

Rats were anesthetized with ketamine (Yuhan, Korea) and xylazine to measure right ventricular systolic pressure (RVSP) 22 days after MCT injection (Bayer Korea, Korea). The pressure transducer was inserted into the RV through the right jugular vein and connected to PowerLab (AD Instruments Pty. Ltd, Australia). Following stabilization, RVSP was measured for 10 min and blood was drawn from the abdominal aorta (27). Hearts and lungs were promptly removed and weighed. The right lungs were dissected and perfused with ice-cold phosphate-buffered saline, then snap-frozen in liquid nitrogen and stored at -80 °C for subsequent biochemical analyses. The left lung was embedded in 10% formalin for histopathological and immunofluorescent staining.

***Electrocardiogram***

Electrocardiogram (ECG) traces were recorded on day 22 before the rats were sacrificed. Needle electrodes were placed under the skin of the animals in the lead II position. ECG parameters were recorded with PowerLabs and LabChart software (ADInstruments, Australia).

***Measurements of cytokine concentrations in lung tissue***

Cytokine levels (IL-1β, TNF-α, IL-6, and NF-κB) were assessed using ELISA kits from ZellBio GmbH (Germany). The quantitative ELISA sandwich technique was employed for quantification in lung tissues. In this method, 100 μl of standard or sample solutions were added to microplate wells and incubated at 37 °C for 2 hr. Biotin-conjugated antibodies specific to the targeted cytokines were added to each well for an additional hour at 37 °C. After washing, avidin conjugated with horseradish peroxidase was added, followed by incubation at 37 °C for 1 hr. A chromogenic substrate solution produced a color proportionate to cytokine concentration. The enzyme-substrate reaction was stopped with a stop solution. Color changes were measured by recording optical density (OD) at 450 nm using a Bio Tek ELISA reader. Cytokine concentrations were determined by comparing sample OD values to a standard curve generated with known standard concentrations (28).

***Measurements of lung tissue oxidants and anti-oxidative enzymes***

The left lung was resected after being washed with saline via the pulmonary artery. A nine-fold volume of phosphate-buffered saline (PBS) was added before gentle grinding at 4 °C. The levels of SOD, CAT, and MDA in lung tissue homogenates were measured using a colorimetric technique and matching kits.

***Measurement of MDA***

The MDA measurements were conducted using a thiobarbituric acid reaction method with the MDA assay kit (Cat No: ZB-MDA) from ZellBio GmbH (Germany). For the analysis, 0.15 ml of the sample was placed in a designated sample tube, 0.15 ml of a standard solution in the standard tube, and 0.15 ml of dehydrated alcohol in the blank testing tube. Then, 4 ml of a mixed reagent was added to each tube. After thorough mixing and incubation at 95 °C for 40 min, the specimens were cooled in running water and centrifuged at 3500 rpm for 10 min. Finally, the supernatant from each tube underwent colorimetric analysis at 532 nm (with zero adjustment using distilled water) (29).

***Measurement of SOD***

SOD activity was determined using the SOD assay kit (Cat No: ZB-SOD) from ZellBio GmbH (Germany) with the WST-1 method. Control wells contained a mixture of double distilled water, enzyme working solution, and substrate application solution. Blank control wells had a mixture of double distilled water, enzyme dilution solution, and substrate application solution. Measurement wells were prepared by combining these mixtures. After incubation at 37 °C, absorbance at 450 nm was measured using a microplate reader (29).

***Measurement of CAT***

In the CAT assay using the ZellBio ELISA kit, assay buffer was added to the wells. A mixture of methanol and the sample was introduced. Diluted hydrogen peroxide was added to initiate enzymatic reactions. The plate was incubated for 20 min at room temperature. The reaction was terminated with potassium hydroxide and catalase periodate. After further incubation, catalase potassium periodate was added. Absorbance was measured at 540 nm (29).

***Measurement of iNOS***

NO levels in lung tissue were measured using an ELISA kit (MyBioSource, Cat No: MBS263618). This competitive enzyme immunoassay method used an anti-iNOS monoclonal antibody and iNOS-HRP conjugate. Lung samples and buffer were incubated with the iNOS-HRP conjugate on a pre-coated plate for one hour. After incubation, wells were washed, and an HRP enzyme substrate was added, resulting in a blue complex. The reaction was stopped with a stop solution, turning the solution yellow, and the yellow color intensity was measured at 450 nm using a microplate reader (30).

***Histopathological studies***

We randomly selected three left lung tissue specimens from each group. These specimens were fixed in 10% neutral buffered formalin for 48 hr, followed by an additional 48-hour immersion in 10% neutral buffered formalin for optimal preservation. The specimens underwent systematic dehydration in graded alcohol concentrations, residual alcohol was removed with xylene treatment, and they were expertly embedded in paraffin. The resulting paraffin blocks were sectioned into 5-μM-thick slices using a microtome, floated on a water bath, mounted on microscope slides, and dried in a 60 °C oven for 20 min. Sections were meticulously stained with hematoxylin and eosin to highlight histopathological features. A blind evaluation was performed on three randomly selected sections from each animal, resulting in a total of 15 images per animal. Numerical results were recorded for histopathological tests to ensure transparency and replicable analysis. Images at 40x magnification were analyzed at five random points within each section using Image J software. This rigorous methodology ensured the accuracy and impartiality of our histopathological assessments.

***MrgD receptor measurement by immunofluorescent staining***

Paraffin-embedded lung tissue sections (5 μm thick) underwent immunofluorescence analysis. The process included deparaffinization, rehydration, and PBS washing. To enhance cell membrane permeability, Triton was applied. Unwanted secondary antibody reactions were prevented by incubating samples in goat serum. Primary antibodies were applied, followed by refrigeration for 24 hr. After primary antibody incubation, samples were washed and secondary antibodies were added, followed by incubation. The samples were washed again and treated with DAPI. Glycerol and PBS were applied for visualization, and samples were examined using fluorescent microscopy. Marker intensity was quantified as a percentage of green color in the image. This rigorous immunofluorescence protocol ensured precise marker assessment in the lung tissue sections.

***Statistical analysis***

The statistical analysis was conducted using two-way analysis of variance (ANOVA), followed by *post hoc* Tukey test to examine the differences between the means of groups. This analysis was performed using SPSS version 27. For generating the figures, GraphPad Prism 7.04 (GraphPad Software, USA) was utilized. The statistical significance threshold was set at *P*<0.05.

## Results

***Hemodynamics***

In our rat model of PAH, MCT injection led to significant hemodynamic changes. RVSP increased to 47.78 ± 1.90 mmHg in the MCT group compared to the control group’s 20.97 ± 1.70 mmHg (*P*<0.001), mitigated by Alamandine co-treatment (MCT+Ala group: 29.26 ± 2.30 mmHg, *P*<0.001).

RV hypertrophy, indicated by the RV/(LV+SP) weight ratio, increased in the MCT group to 0.41 ± 0.04 (*P*<0.001), significantly higher than the control group’s 0.22 ± 0.02. The MCT+Ala group showed a moderate ratio of 0.33 ± 0.03 (*P*<0.001).

Body weight in the MCT group decreased significantly to 218.00 ± 12.30 g, compared to the control group’s 326.00 ± 14.57 g (*P*<0.001). Alamandine co-administration mitigated this loss, with the MCT+Ala group at 260.00 ± 21.13 g.

The RVW/BW ratio in the MCT group was substantially higher at 1.22 ± 0.09 g/kg compared to the control group’s 0.53 ± 0.04 g/kg (*P*<0.001), moderated in the MCT+Ala group (0.87 ± 0.03 g/kg).

The lung W/BW ratio in the MCT group significantly increased to 6.18 ± 0.20 compared to the control’s 3.69 ± 0.10 (*P*<0.01), with alamandine co-treatment mitigating this rise in the MCT+Ala group (4.82 ± 2.30, *P*<0.001, Table 1). 

***Electrocardiogram***

MCT administration led to various electrocardiographic changes, including a significant decrease in heart rate, with the MCT group averaging 248.30 ± 60.70 beats/minute, contrasting the control group’s 373.80 ± 53.70 beats/minute (*P*<0.05). Alamandine co-treatment moderated this decline, resulting in a mean rate of 332.60 ± 98.90 beats/minute in the MCT+Ala group, though this change wasn’t statistically significant.

The P-R interval in the control group averaged 48.80 ± 5.80 ms, while MCT treatment reduced it to 45.40 ± 11.80 ms. Co-treatment with alamandine somewhat restored this, resulting in an average of 47.30 ± 10.30 ms for the MCT+Ala group, with no statistically significant variances (*P*>0.05).

**Figure 1:**
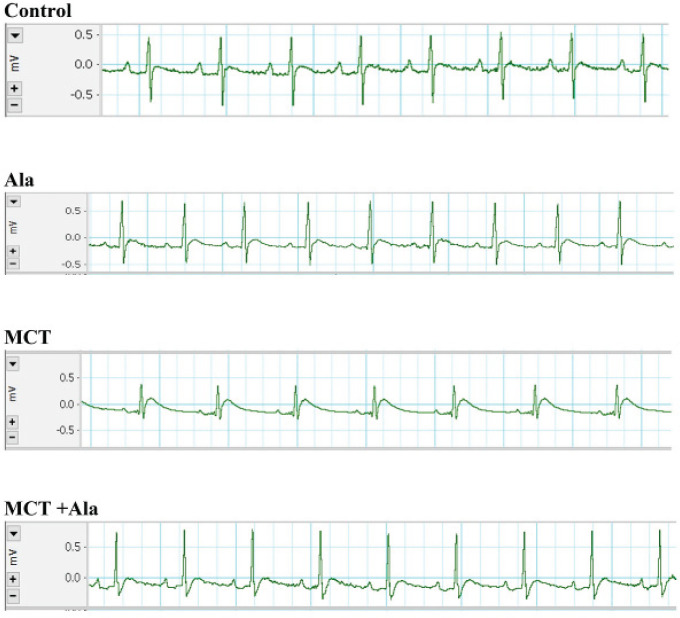
Electrocardiogram recordings from anesthetized rats across different experimental groups, comprising the control group, monocrotaline (MCT)-treated group, alamandine group, and MCT + alamandine group

MCT significantly reduced the QRS amplitude, with the control group at 1.09 ± 0.14 mv and the MCT group at 0.63 ± 0.17 mv (*P*<0.001). Alamandine co-treatment moderated this effect, with the MCT+Ala group exhibiting an amplitude of 1.04 ± 0.16 mv, a significant rebound from the MCT-only group (*P*<0.001). Additional details and visual representation can be found in Table 2 and Figure 1.

***Effect of co-administration of alamandine and MCT on oxidative stress markers (MDA, SOD, and catalase) in lung tissue***

MCT administration significantly increased lung MDA levels (16.82 ± 4.92 µM) compared to the control group (2.88 ± 1.79 µM, *P*<0.001), while co-treatment with alamandine reduced MDA levels (0.96 ± 0.23 µM) (*P*>0.05). 

MCT administration led to a notable reduction in SOD levels (13.03 ± 5.93 IU/ml) compared to the control group (36.32 ± 12.11 IU/ml, *P*<0.001). Co-treatment with alamandine increased SOD levels (85.33 ± 12.81 IU/ml) (*P*<0.001).

**Figure 2:**
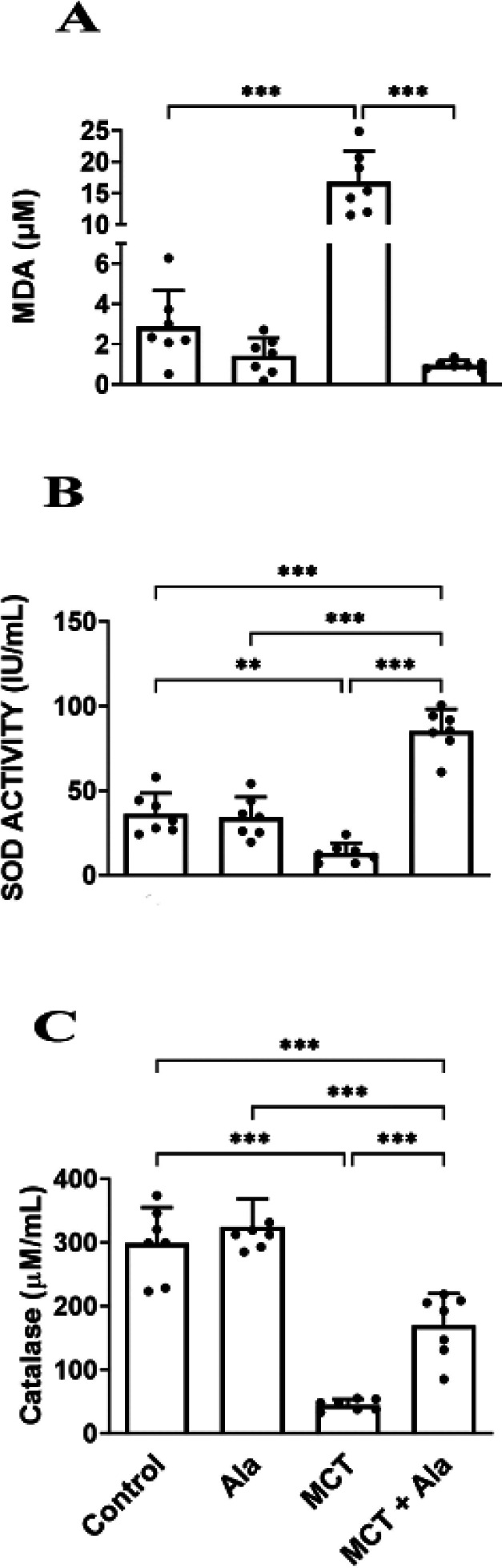
Oxidative stress markers in rats with monocrotaline (MCT)-induced pulmonary hypertension

Catalase levels significantly decreased in lung tissue with MCT administration (44.88 ± 8.74 µM/ml) compared to the control group (298.70 ± 56.11 µM/ml, *P*<0.001). Co-treatment with alamandine partially reversed this effect (169.90 ± 49.73 µM/ml) (*P*>0.05) (Figure 2).

***Effect of co-administration of alamandine and MCT on iNOS in lung tissue***

**Figure 3:**
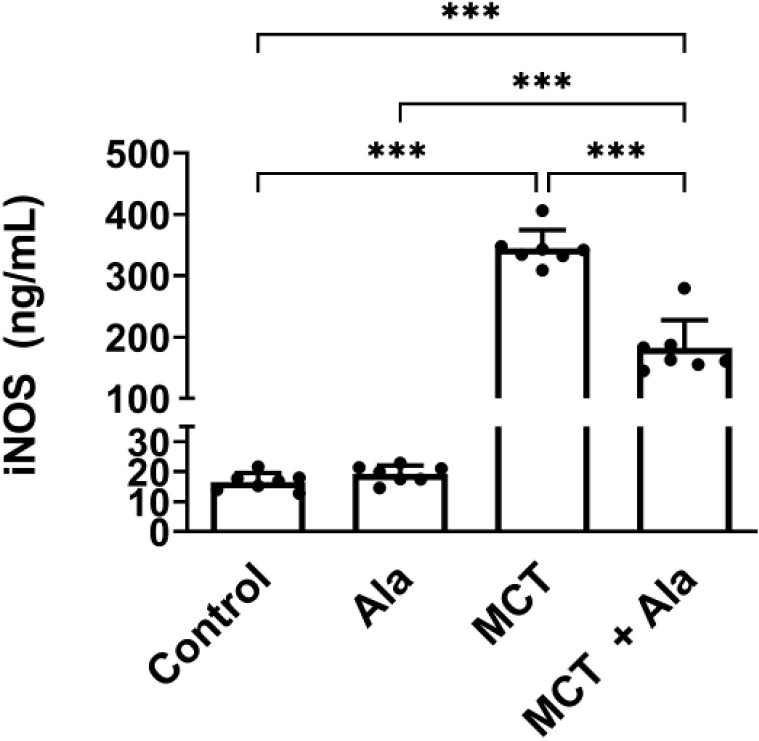
Inducible nitric oxide synthase (iNOS) expression in rats with monocrotaline (MCT)-induced pulmonary hypertension (data presented as mean ± SD, n=7)

MCT administration significantly increased iNOS levels to 345.10 ± 29.78 ng/ml compared to the control group’s 16.60 ± 2.95 ng/ml (*P*<0.001). Alamandine treatment reduced iNOS levels to 182.10 ± 45.65 ng/ml in the MCT+Ala group (*P*<0.001) (Figure 3).

***Effect of co-administration of MCT and alamandine on inflammatory cytokines***

MCT significantly increased TNF-α levels (234.50 ± 51.91 pg/ml) compared to the control group (41.29 ± 6.47 pg/ml, *P*<0.001). Co-administration with alamandine lowered TNF-α levels to 109.50 ± 18.12 pg/ml in the MCT+Ala group(*P*<0.001).

MCT administration raised NF-κB levels (16.82 ± 4.92 pg/ml) compared to the control group (2.88 ± 1.79 pg/ml, *P*<0.001). Co-administration with alamandine mitigated this increase to 0.96 ± 0.23 pg/ml in the MCT+Ala group (*P*<0.01).

For IL-6, MCT significantly increased levels (71.36 ± 4.73 pg/ml, *P*<0.001), which were moderated by alamandine to 26.88 ± 9.60 pg/ml in the MCT+Ala group(*P*<0.001).

**Figure 4.**
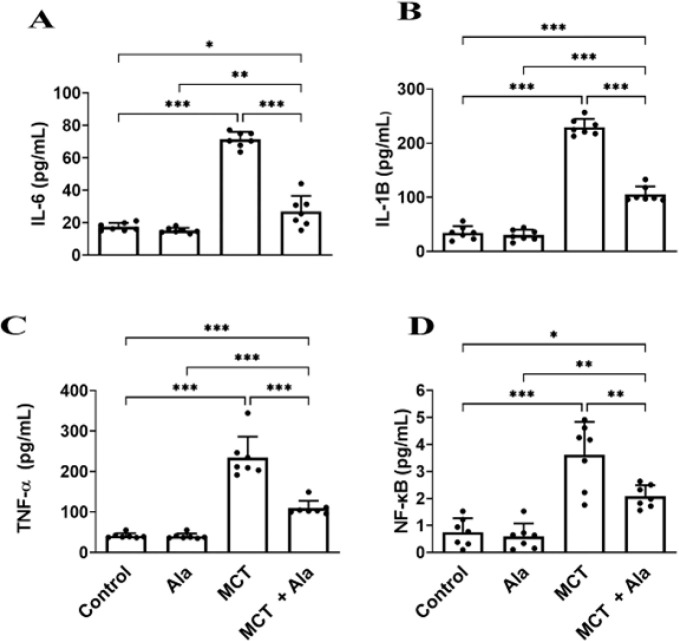
Inflammatory cytokines in rats with monocrotaline (MCT)-induced pulmonary hypertension

IL-1β levels surged with MCT administration (229.60 ± 15.25 pg/ml, *P*<0.001) and were reduced to 105.30 ± 14.50 in the MCT+Ala group (*P*<0.001) (Figure 4).

***Histopathological findings***

Figure 5 shows a cross-section of rat lung tissue stained with hematoxylin and eosin. Based on the obtained results, it was found that the amount of inflammation, secretion of mucus, bleeding inside the lung tissue, and secretion from inflammatory cells in the MCT group were higher than in other groups. In addition, the results showed that the amounts of these evaluated factors were high in the (MCT + Ala) group but lower than in the MCT group. Examining the images obtained from the control group showed ciliated cells with a normal appearance in bronchiole tissues. Air sacs with a pneumocyte cell layer were observed. In this row of cells, type 1 alveolar cells, which are very thin squamous cells, covered 95% of the alveolar surface and created a barrier between the air and the alveolar wall. Also, type 2 alveolar cells, which are cells that secrete surfactant, were observed normally in this group. In general, the tissue was completely normal without any thickening or signs of cellular metaplasia. The images related to the group (MCT + Ala) showed that the bronchioles in this group had a normal appearance. Among the cells, bleeding effects were greatly reduced compared to the MCT group (Figure 5). 

**I** ***mmunofluorescence for MrgD Receptors in lungs***

**Figure 6:**
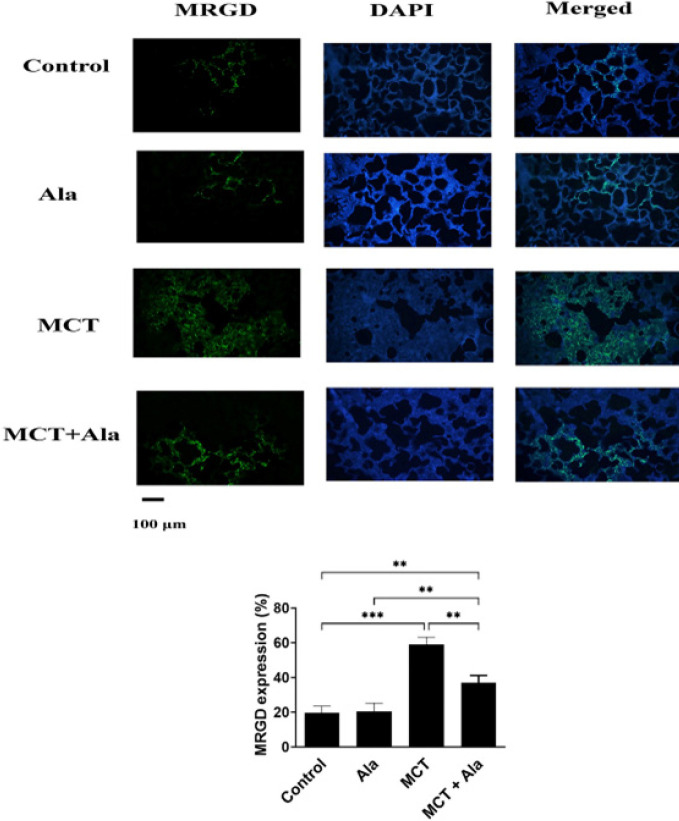
Immunofluorescence detection of Mas-related G protein-coupled receptor member D (MrgD) in the lungs of rats

The immunofluorescence staining of MrgD receptors was significantly increased in the lung tissue of MCT-treated rats compared to those from control rats (*P*<0.001). Alamandine treatment significantly decreased MrgD receptor levels compared with the MCT group (*P*<0.01) (Figure 6).

**Table 1:** Comparison of right ventricular systolic pressure (RVSP), right ventricle to (left ventricle + septum) ratio (RV/(LV+SP) ratio), right ventricular weight to body weight ratio (RVW/BW), lung weight to body weight ratio (lung W/BW ratio), and body weight (BW) in monocrotaline-induced pulmonary hypertensive rats treated with alamandine, compared to the control group

	Control	Ala	MCT	MCT+Ala
RVSP (mmHg)‎	‎20.97±1.7	‎22.19±1.2	‎47.78±1.9‎^***^^‎^	‎29.26±2.3‎^***^^‎‎^^###^^‎^
BW (g)‎	‎326±14.57	‎346± 15.9‎	‎218.0±12.3^***^^‎^‎	‎260.±21.13^***###^^‎^
RVW/(LV+SP) ‎ratio	‎0.22±0.02	‎0.21±0.01	‎0.41±0.04‎^***^^‎^	‎0.33±0.03^***^^‎^^###^
RVW/BW (g/kg)‎	‎0.53±0.04	‎0.58±0.07^‎^	‎1.22±0.09‎^***^^‎^	‎0.87±0.03^***##^
Lung W/BW ratio (mg/g)	‎3.69±0.1‎	‎3.53±0. 6	‎6.18±0.2‎‎^**^	‎4.82±0. 1^*^^‎^^##^^‎‎^

The data are presented as mean ± standard deviation (SD); n=7 for each treatment group.

*P<0.05, **P<0.01, ***P<0.001 indicate statistical significance compared to the control group

#*P*<0.05, ##*P*<0.01, ###*P*<0.001 indicate statistical significance compared to the monocrotaline (MCT) group

Notably, the MCT-treated group exhibited significant reductions in heart rate, and QRS amplitude, which were effectively mitigated by alamandine.

Ala: Alamandine; MCT: Monocrotaline

**Table 2:** Assessment of electrocardiographic parameters in various experimental groups: Comparative analysis of heart rate, P-R interval, and QRS amplitude in control, alamandine-administered, monocrotaline (MCT)-treated, and MCT + alamandine-treated rats

Title 1	Control	Ala	MCT	MCT+Ala
HR (bpm)	373.8±53.7	361.1±58.2	248.3±60.7 ^*^	332.6±98.9
P-R interval (ms)	48.8±5.8	49.2±6.8	45.4.7±11.8	47.3±10.3
QRS amplitude (mv)	1.08±0.14	‎1.11±0.15‎	0.63±0.17^***^	1.04±0.16^###^

Data are expressed as mean ± SD; n = 7 for each group

Ala: Alamandine; MCT: Monocrotaline; HR:Heart rate; bmp: Beat per minute; ECG: Electrocardiogram

*P<0.05, ***P<0.001 compared to the control; ###*P*<0.001 compared to the MCT group.

(A) Malondialdehyde (MDA) levels in lung tissue, (B) Superoxide dismutase (SOD) activity in lung tissue, and (C) Catalase activity in lung tissue (data presented as mean ± SD, n=7). Statistical comparisons were performed using a two-way analysis of variance (ANOVA) followed by Tukey's post hoc test.

The significance levels are represented as *P<0.05, **P<0.01, and ***P<0.001.

Statistical comparisons were conducted using a two-way analysis of variance (ANOVA) followed by Tukey's post hoc test.

The significance levels are represented as *P<0.05, **P<0.01, and ***P<0.001.

(A) Interleukin 6 levels in lung tissue, (B) Interleukin-1 beta (IL-1β) levels in lung tissue, (C) Tumour necrosis factor-alpha (TNF-α) levels in lung tissue, and (D) Nuclear factor kappa B (NF-κB) cell content in lung tissue (data presented as mean ± SD, n=7). Statistical comparisons were performed using two-way analysis of variance (ANOVA) followed by Tukey's *post hoc* test.

Significance levels were denoted as **P<0.01, ***P<0.001, and *P<0.05.

**Figure:**
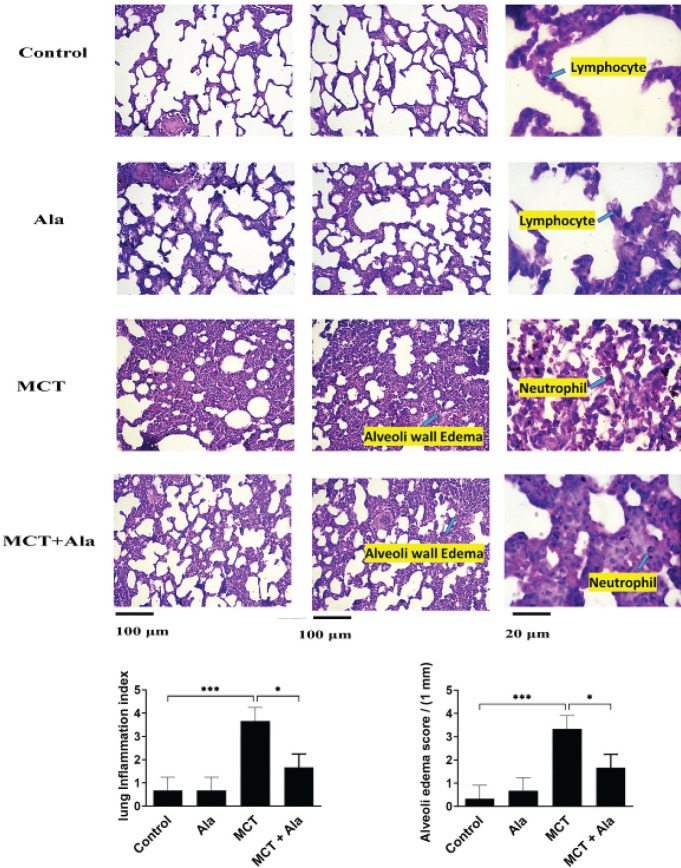
**Figure 5**Histological sections of lung tissue from rats in the following groups: control group, monocrotaline (MCT)-treated group, alamandine group, and MCT + alamandine group

Hematoxylin and eosin staining at ×100 magnification (data presented as mean ± SD). The significance levels were denoted as **P<0.01, ***P<0.001, and *P*<0.05.

Triple immunostaining was performed to visualize the MrgD receptor (green) and nucleus with DAPI (blue). Representative images showed the expression of MrgD in the lung membrane. Quantitative analysis revealed a higher expression of MrgD in the monocrotaline (MCT)-induced pulmonary hypertension group compared to the control group. However, a decreased expression of MrgD was observed in the MCT + alamandine group compared to the MCT group. The significance levels were denoted as **P<0.01, ***P<0.001, and *P*<0.05.

**Figure 7:**
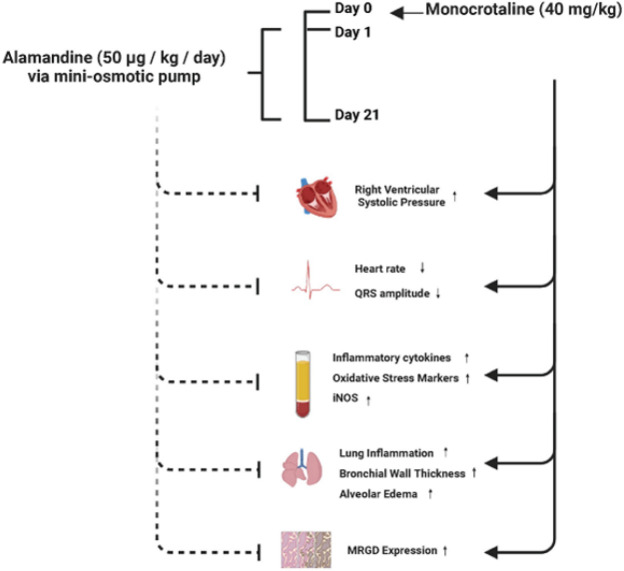
Comprehensive overview of alamandine’s impact on pulmonary hypertension: Attenuation of doxorubicin-induced cardiac contractility reduction, ECG abnormalities, elevated inflammatory cytokines, and lung inflammation

## Discussion

In the present study, we showed that administration of alamandine had protective effects on MCT-induced PAH in rats. These effects could be linked to the reduction in oxidative stress and inflammatory cytokine levels. These positive effects have been discussed previously (31, 32)defined as a mean pulmonary artery pressure by right-sided heart catheterization of at least 25 mm Hg at rest. It is classified into 5 general groups based on the underlying cause, with left ventricular failure and chronic obstructive pulmonary disease being 2 of the most common causes in the United States. Although the specifics of the pathophysiology will vary with the cause, appreciating the risks of pulmonary hypertension and right ventricular failure is critical to appropriately evaluating and resuscitating pulmonary hypertension patients in the emergency department (ED. PAH is characterized by undesirable hemodynamic changes in pulmonary arteries. These changes give rise to a significant increase in PAH and RVSP along with RVH. In animal models, similar changes, RVSP and RVH are commonly considered evidence of the presence of PAH (33, 34). 

The major findings of this investigation are that alamandine suppressed inflammation, reduced reactive oxygen species, and restored injuries to lung tissue caused by oxidative stress. This study showed that alamandine ameliorated PAH, as demonstrated by the improved right ventricular systolic pressure and resolved RV/(LV+SP) weight ratio. Compared with the MCT group, the rats that received alamandine therapies showed improvements in the right hemodynamic parameters (i.e., RVSP). It has been shown that alamandine induces vasodilatory and anti-hypertensive effects through the MrgD receptor (35) and that the reduction of RVSP is probably due to a decline in afterload produced by alamandine action (34). MCT exerted a significant decrease in final body weight, which is a sign of heart failure. This phenomenon is similar to cachexia in chronic heart failure patients. The growth restriction has been reported to be induced by MCT administration; in a previous study, rats became severely anorexic upon exposure to MCT (36). Body weight loss has also been shown to accompany a significant increase in RV/body weight and RV/LV+S ratios, indicating RVH. The observed increase in the lung-to-body weight ratio within the MCT group can potentially serve as an indicator of extensive pulmonary proliferative response (37). However, due to the concurrent reduction in body weight attributed to the effects of monocrotaline, this parameter may not serve as a precise indicator.

In the MCT-treated group, significant reductions in heart rate, QRS amplitude, and duration were observed. The decreases in HR and QRS amplitude could be a result of down-regulation of the b1-adrenoceptor and desensitization of the b1-adrenoceptor-G protein-adenylyl cyclase system due to MCT’s effect, followed by RVH (38).Moreover, MCT’s direct cardiotoxic action and coronary artery medial wall-thickening effect could influence the depression of ventricular function (22). Also, RVH may contribute to the impairment of electromotive forces via a reduction in mean QRS vector magnitude (39). When treatment was combined with alamandine, RV/body weight and lung/body weight ratios normalized, and the RV/LVS ratio significantly decreased compared to the MCT group. QRS amplitude was also normalized.

Three main factors are known as contributors to the development and progression of endothelial injury in PAH, namely, inflammation, NO, and oxidative stress. Inflammation is considered to be a major factor in initiating and maintaining vascular remodeling in PAH animal models (40). Consequently, reducing the inflammatory response might ameliorate PAH development. Previous studies have indicated that the TNF-α level in heritable and idiopathic PAH patients was considerably higher than in healthy individuals (41, 42). Our data demonstrated statistically significant reductions in TNF-α, IL-1β, and IL-6 levels (*P*<0.001), as well as NF-κB levels (*P*<0.05), in the almandine-treated group compared to the MCT-induced PAH group. These findings indicate that alamandine counteracts the inflammatory response in MCT-induced PAH rats.

NO, produced by the nitric oxide synthases (NOSs) family, is an often-recognized key factor contributing to the development and progression of endothelial injury in PAH. Within this family, iNOS stands out as an inducible member that generates a sustained production of NO after induction (43). The course of PAH induced by MCT injection is progressive, with persistent inflammation defined by an acute phase within the first 6 days after MCT treatment, followed by a chronic phase (44). There is no or little expression of iNOS in normal tissue; obvious iNOS expression can occur only in cases of acute or chronic inflammation (45). MCT treatment overactivated the NO signaling pathway. The activation of iNOS generated a large amount of NO, which, in turn, contributed to a further deterioration of the vascular endothelial cells (46). In this study, MCT significantly increased iNOS, while co-administration of alamandine and MCT decreased NOS. This reduction could be a result of a decrease in the inflammatory factors associated with alamandine.

A study demonstrated that both SOD and glutathione peroxidase activities decreased in PAH lungs when compared to healthy controls (47). Furthermore, Irodova *et al*. showed an increase in the level of MDA in PAH patients in comparison to healthy controls (48). This elevation was more prominent in patients with severe pulmonary hypertension, while the results of glutathione peroxidase activity were inconclusive. In this study, we discussed three well-studied oxidative stress biomarkers in tissues (i.e., CAT and SOD enzyme activity), as well as MDA levels. The first two biomarkers represent the core of the enzymatic antioxidant system. Generally, SODs catalyze the conversion of superoxide radicals to H_2_O_2_, which, in turn, is converted into water by CAT. As shown *in vivo*, SOD and CAT mimetics can reverse pulmonary vascular remodeling and PAH. Therefore, it is likely that these enzymes’ activities have pathologic values in PAH patients (48). MDA can be a marker for lipid peroxidation and can be used to indirectly assess the intensity of damage to cell membranes (49). In our study, the MDA levels were significantly decreased as a result of co-administration of alamandine and MCT. On the other hand, levels of SOD and CAT enzyme activity increased. The results of our experiment are consistent with those of a previous study in which alamandine increased antioxidant protein expression in ventricles exposed to I/R injuries (50). Moreover, inhibiting the PKC/reactive oxygen species signaling pathway reduced renal damage in Dahl rats (51).

In this study, we also investigated the expression of MrgD receptors. Alamandine performs its actions by binding to the MrgD receptor (16, 25, 35, 52). We found that MrgD expression was higher in the lungs of the MCT group than in the group that received the co-administration of alamandine and MCT, suggesting that alamandine receptor activity is enhanced in the lungs during pulmonary hypertension. Previously reported studies have indeed shown an up-regulation of MrgD expression in various hypertensive conditions (53). For instance, the MrgD expression is increased in the heart of spontaneously hypertensive rats (54) and ventricular myocytes from the TGR (mREN2)27 rat model of hypertension (55). This up-regulation under pathological scenarios underscores the potential protective role MrgD might play during the course of disease progression (53). Importantly, our study further explores this avenue by demonstrating that alamandine treatment leads to a reduction in MrgD receptor levels, which is concomitant with decreased pulmonary arterial pressure. This implies a mechanistic link between MrgD receptor modulation and PAH. The relationship between alamandine, MrgD receptors, and pulmonary arterial pressure offers a promising avenue for therapeutic interventions and warrants further investigation.

These results provide evidence that alamandine may be a protective agent in the treatment of pulmonary hypertension. Furthermore, our findings are effectively summarized in the graphical abstract (Figure 7), which provides a visual representation of the key results and insights presented in this study.

## Conclusion

Our study demonstrated that alamandine effectively mitigated MCT-induced PAH in rats. It achieved this by reducing oxidative stress, inflammation, regulating inducible nitric oxide synthase, and ameliorating histopathological changes. The observed elevation in MrgD expression in the context of PAH may have signified a compensatory mechanism initiated to counteract the effects of PAH and potentially restore pulmonary homeostasis. Notably, alamandine treatment, by reducing PAH, may have counteracted this increase in MrgD expression, highlighting its role in regulating this compensatory response. These findings underscored the therapeutic promise of alamandine in managing PAH.

## Authors’ Contributions

A SH and K J conceived the study and designed the experiments. F A and A SH were responsible for conducting the research. A SH and K J performed data analysis and also wrote the manuscript. All authors have thoroughly reviewed and approved the final version of the manuscript.

## Funding

This study was funded by Fasa University of Medical Sciences, Iran (grant number 400303).

## Ethics Commitee Approval

Animals were approved by Fasa University of Medical Sciences with the ethical code(IR.FUMS.AEC.1401.010), obtained on September 7, 2022.

## Conflicts of Interest

The authors declare that they have no conflicts of interest concerning the research, authorship, and/or publication of this article.
